# Processing and Real-Time Monitoring Strategies of Aflatoxin Reduction in Pistachios: Innovative Nonthermal Methods, Advanced Biosensing Platforms, and AI-Based Predictive Approaches

**DOI:** 10.3390/foods14193411

**Published:** 2025-10-02

**Authors:** Seyed Mohammad Taghi Gharibzahedi, Sumeyra Savas

**Affiliations:** 1 Institute of Materials Science, Faculty of Engineering, Kiel University, 24143 Kiel, Germany; 2 Biosensors and Biotechnology Laboratory (BBL), Medical School, Bandırma Onyedi Eylül University, Bandırma 10200, Balıkesir, Türkiye; ssavas@bandirma.edu.tr

**Keywords:** pistachio nuts, aflatoxin B_1_, *Aspergillus flavus*, mycotoxin determination, cold plasma, biosensors, smart sensing systems, predictive modeling, artificial intelligence, machine learning

## Abstract

Aflatoxin (AF) contamination in pistachios remains a critical food safety and trade challenge, given the potent carcinogenicity of AF-B_1_ and the nut’s high susceptibility to *Aspergillus* infection throughout production and storage. Traditional decontamination methods such as roasting, irradiation, ozonation, and acid/alkaline treatments can reduce AF levels but often degrade sensory and nutritional quality, implying the need for more sustainable approaches. In recent years, innovative nonthermal interventions, including pulsed light, cold plasma, nanomaterial-based adsorbents, and bioactive coatings, have demonstrated significant potential to decrease fungal growth and AF accumulation while preserving product quality. Biosensing technologies such as electrochemical immunosensors, aptamer-based systems, and optical or imaging tools are advancing rapid, portable, and sensitive detection capabilities. Combining these experimental strategies with artificial intelligence (AI) and machine learning (ML) models can increasingly be applied to integrate spectral, sensor, and imaging data for predicting fungal development and AF risk in real time. This review brings together progress in nonthermal reduction strategies, biosensing innovations, and data-driven approaches, presenting a comprehensive perspective on emerging tools that could transform pistachio safety management and strengthen compliance with global regulatory standards.

## 1. Introduction

**Figure 1:**
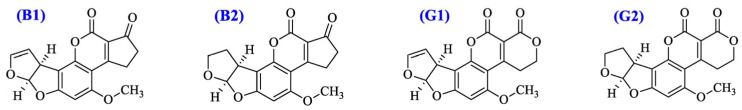
Molecular structures of the most prevalent AFs (B_1_, B_2_, G_1_, and G_2_) in pistachios (reprinted with permission from He et al. [1]. Copyright© (202)] Elsevier).

Pistachios (*Pistacia vera* L.) are a high-value nut commodity widely consumed worldwide for their nutritional and health benefits, yet they are also among the most susceptible to contamination by mycotoxins, especially aflatoxins (AFs). The most common AFs are AFB_1_, AFB_2_, AFG_1_, and AFG_2_, which are among the more than 20 types identified to date (Figure 1) [1]. These mycotoxins, particularly AFB_1_, which are significant food safety risks, are mainly produced by *Aspergillus flavus* and *A. parasiticus* [2,3]. Surveys indicate that pistachios are consistently at the top of tree nuts in terms of AF contamination levels, largely due to shell splitting during maturation, insect infestation, and unfavorable postharvest handling [4,5]. A systematic review of Iranian pistachios, one of the main global exports, showed that the presence of AFB_1_ in ~72% of reported cases was over the Iranian national limit of 5 µg/kg. Also, the concentration range varied from <0.066 µg/kg up to 1485 µg/kg depending on cultivar and handling conditions [3]. These findings reveal pistachios’ status as a high-risk nut for AF contamination and a major contributor to dietary AF exposure worldwide [2,3].

Critical pre- and postharvest factors drive pistachio contamination dynamics. Early splitting or hull cracking significantly increases fungal penetration and toxin production, while delays in hulling and drying escalate contamination. For example, in Iran’s Ohadi pistachio cultivar, a 48 h delay in processing increased AF levels up to 66.1 ppb, compared to only 0.1 ppb when hulling was immediate [4]. Similarly, the insect damage amplifies susceptibility by creating entry points for *Aspergillus* spp. [5]. Outbreak notices from the Rapid Alert System for Food and Feed (RASFF) always highlight pistachios exported from Turkey, Iran, and the USA as frequent sources of AF violations in the European Union (EU) market [2]. Thus, beyond its toxicological implications, AF contamination in pistachios is also an economic barrier, as shipments exceeding maximum levels are subject to rejection, resulting in substantial losses for producers and exporters. These realities make pistachio AF control not only a food safety imperative but also a trade and regulatory challenge that motivates the exploration of innovative processing, monitoring, and predictive strategies [6,7,8].

Processing interventions to reduce AFs in pistachios span from conventional postharvest decontamination (i.e., mechanical separation, thermal treatments, and irradiation) to innovative nonthermal approaches. Mechanical sorting, optimized drying, and irradiation are not only considered baseline tools, but urge the adoption of sustainable options that preserve sensory, textural, and nutritional quality [9]. In practice, integrated systems in U.S. tree nuts pair orchard hygiene and insect control with postharvest segregation and rapid removal of suspect kernels. These steps directly lower AF load entering processing and complement downstream decontamination [10]. Online fluorescence spectroscopy enables real-time detection and sorting of AFB_1_-contaminated pistachio kernels at industrial throughputs (≈1 kernel s^−1^) with discrimination down to 5 ppb, providing a practical processing-line barrier to contaminated units [11]. A leading nonthermal decontamination candidate is cold atmospheric plasma (CAP), which in pistachios has demonstrated inactivation of *A. flavus* and reduction in total AFs while maintaining product quality attributes, positioning it as a greener replacement or adjunct to heat- or chemical-based steps [6]. Although chiefly preharvest-oriented, targeted chemical control with selected fungicides such as prochloraz, pyrimethanil, or cyprodinil + fludioxonil has shown up to 99.9% inhibition of AF production in multi-year orchard trials, informing integrated programs that couple field suppression with postharvest sorting and nonthermal processing to minimize residual risk [12]. Overall, nonthermal technologies such as irradiation, ozone, UV-based treatments, pulsed light, high-pressure processing, ultrasound, and cold plasma show strong potential to inactivate *A. flavus* and degrade AFs in pistachios while better preserving product quality [13,14,15].

Real-time monitoring of AFs in pistachios has gained increasing attention due to the limitations of conventional methods (e.g., high-performance liquid chromatography (HPLC) and enzyme-linked immunosorbent assay (ELISA)), including high costs, being time-consuming, and the need for specialized laboratories [16,17]. In addition to conventional chromatography- or immunoassay-based techniques, rapid and non-destructive methods for aflatoxin detection are increasingly gaining attention. Terahertz (THz) spectroscopy has emerged as a powerful tool for sensitive and quantitative analysis. Ge et al., demonstrated that chemometric models combined with THz time-domain spectroscopy (THz-TDS) could reliably determine AFB_1_ concentrations in acetonitrile, highlighting the potential of THz-based approaches for food safety monitoring [18]. More recently, Hu et al., reported a metamaterial-enhanced THz sensor capable of highly sensitive detection of AFB_2_ solutions, achieving limits of detection (LODs) down to the order of 10^−11^ mg/mL [19]. These advances illustrate that THz spectroscopy, especially when coupled with machine learning (ML) or metamaterial design, can complement modern biosensing platforms by providing rapid, non-destructive, and ultra-sensitive detection of aflatoxins in food matrices.

Different biosensing platforms have emerged as powerful alternatives for on-site and rapid detection. Electrochemical immunosensors employing antibody–antigen interactions can detect AFB_1_ at very low concentrations with high selectivity [20]. DNA-based biosensors, targeting AF biosynthetic genes in *A. flavus*, provide sensitive detection of toxigenic strains before toxin accumulation occurs [21]. Advanced nanomaterial-based sensors, such as metal–organic frameworks (MOF)/MXene composites, offer ultra-low detection limits down to the picogram level and strong reproducibility, enabling application in real pistachio samples [22]. Cell-based biosensors using membrane-engineered mammalian cells with anti-AFB_1_ antibodies allow direct recognition of toxin activity in contaminated samples [23]. In addition, intelligent, non-destructive approaches such as electronic nose systems combined with machine vision can discriminate AF-producing fungal contamination in pistachios at early stages [24]. By and large, these biosensing strategies, including immunosensors, aptasensors, DNA sensors, cell-based assays, and multi-sensor systems, demonstrate remarkable potential for real-time AF surveillance in pistachios, revealing compliance with strict food safety regulations.

Artificial intelligence (AI) and ML are basic data-driven approaches for predictive modeling of AF contamination in pistachios. Mechanistic models such as AFLA-PISTACHIO have demonstrated the feasibility of forecasting AFB_1_ risk using weather-driven and crop phenology data, providing decision support for growers at preharvest stages [25]. Accordingly, ML approaches coupled with hyperspectral imaging (HSI) have achieved high classification accuracy in distinguishing contaminated pistachios, facilitating non-destructive and real-time detection [26]. Broader AI frameworks have also been applied to predict fungal growth and mycotoxin biosynthesis under different environmental and storage conditions, signifying their capacity to guide intervention strategies [27]. These predictive tools complement modern regulatory frameworks for mycotoxins in ready-to-eat foods, presenting faster and more adaptive monitoring than conventional assays [28]. Contemporary analytical methods like surface-enhanced Raman scattering (SERS) integrated with supervised learning further improve stability, reproducibility, and sensitivity in AFB_1_ quantification [29]. Also, innovative technologies including HSI, luminescent metal–organic frameworks, and AI-driven machine vision promise scalable, non-intrusive monitoring throughout the pistachio supply chain [30]. Hence, these advances show the importance of AI and ML in safeguarding consumer health, supporting international trade compliance, and decreasing economic losses linked to AF contamination.

To the best of our knowledge, there is no comprehensive review to address the integration of nonthermal treatments, modern biosensing platforms, and AI- and ML-based predictive models for the mitigation and monitoring of AFs in pistachio nuts. Therefore, the present study aims to overview new achievements in these three complementary domains, emphasizing their synergistic potential to reduce contamination risk, enable real-time and non-destructive detection, and strengthen predictive food safety assurance. By consolidating current knowledge and identifying research gaps, this work seeks to provide a framework for the development of integrated, sustainable strategies that ensure the safety, quality, and global trade compliance of pistachios.

## 2. Aflatoxin Contamination in Pistachios

### 2.1. Sources

AFs in pistachios are produced mainly by *A. flavus* and *A. parasiticus* that colonize nuts both in the orchard and after harvest. Major preharvest drivers are early hull-split/shell cracking, insect injury (such as navel orangeworm (*Amyelois transitella*)), drought or heat stress, and warm–humid orchard microclimates during nut maturation [10]. Harvest/postharvest drivers include delays in hulling/drying, insufficient drying (kernel moisture/water activity remaining high), warm–humid storage, and poor aeration or hygiene of bins/equipment, all of which accelerate fungal growth and AF biosynthesis [3,10]. For instance, a 48 h delay in processing Ohadi pistachios elevated AF from ~0.1 to ~66 µg/kg [4]. Across supply chains, lot mixing, transport under humid conditions, and inadequate sorting allow localized contamination to propagate into consignments [2,11].

### 2.2. Health Risks and Acceptable Levels

The International Agency for Research on Cancer (IARC) has classified AFB_1_ as a Group 1 carcinogen. This intake of AFB_1_ is strongly associated with the high risk of hepatocellular carcinoma, especially in people infected with hepatitis B. It is also associated with immune suppression and impaired growth in children. In addition, cases of acute aflatoxicosis have been documented, some of which have been fatal [28]. Because AFB_1_ is genotoxic and carcinogenic, agencies avoid setting a tolerable daily intake and instead apply margin-of-exposure or cancer-potency approaches [27]. To reduce these risks, strict regulatory limits on AFs in pistachios and other nuts are enforced worldwide. These measures are designed to minimize dietary exposure and to ensure consumer safety across different populations [2,3,28]. Co-contamination with other mycotoxins, such as ochratoxin A and fumonisins, has also been reported, raising concerns of additive or synergistic effects [27,29].

### 2.3. Regulatory Alerts and Trade Issues

Pistachios remain one of the most frequently flagged nuts for AF contamination in the EU-RASFF. A ten-year survey (2011–2021) showed that pistachio notifications accounted for almost 28% of all nut mycotoxin entries, with consignments from Iran, Turkey, and the United States being the most frequently implicated. Broader reviews of RASFF border rejection data from 2008 to 2023 confirm the recurrent pattern of AF-related rejections of pistachio shipments, although exact counts vary depending on the classification of alerts and the period analyzed. More recent reports continue to list individual alerts in 2023–2024, underlining that pistachio consignments remain under intense scrutiny at EU borders. These notifications highlight that pistachio trade is highly vulnerable to regulatory enforcement and that shipments not conforming to maximum levels are at high risk of rejection in key international markets [30,31,32].

### 2.4. Global Limits and Economic Implications

Regulatory authorities worldwide have established specific AF concentrations in pistachios to safeguard consumers as well as harmonize trade. In the EU, AFB_1_ and total AFs of ready-to-eat pistachios may not exceed 2 µg/kg and 4 µg/kg, respectively. Nonetheless, consignments “subject to sorting/physical treatment” are permitted up to 8 µg/kg AFB_1_ and 10 µg/kg total AFs [2,25,28]. The U.S. Food and Drug Administration (FDA) implements a total AF limit of 20 µg/kg [2,33]. In Iran and China, the maximum permitted level is 5 µg/kg AFB_1_ [3,28]. India currently applies 10 µg/kg AFB_1_ and 15 µg/kg total AFs for nuts, whereas Turkey and the Persian Gulf Cooperation Council (PGCC) countries generally allow 8–10 µg/kg AFB_1_ and 10–15 µg/kg total AFs in pistachios intended for direct consumption. The Codex Alimentarius provides a harmonized reference value of 10 µg/kg total AFs for tree nuts in international trade [28,31,34]. Such regulatory heterogeneity has direct consequences for technology adoption. Exporters targeting the highly stringent EU market must employ more robust or combined mitigation strategies (e.g., CAP or advanced optical sorting) to consistently meet the 2 µg/kg AFB_1_ threshold, whereas the comparatively higher U.S. limit of 20 µg/kg allows reliance on less intensive or lower-cost measures (e.g., conventional ozone treatment). For producers in Iran and Turkey, whose exports frequently face RASFF notifications, aligning with EU standards has become essential to reduce costly rejections. Thus, the variability in global limits not only creates trade barriers but also shapes industry incentives, driving research and adoption of technologies capable of meeting the strictest standards. Despite these regulatory frameworks, pistachios remain among the most rejected nut commodities in international trade due to AF non-compliance. Such rejections impose significant costs related to testing, disposal, and market losses. Global estimates suggest that AF contamination in nuts can result in annual economic losses reaching hundreds of millions of dollars, particularly affecting major exporters such as Iran, Turkey, and the U.S. [10,30].

Beyond maximum limits, the adoption of emerging mitigation technologies is constrained by both regulatory and economic barriers. Since exporters must meet the strictest limits (e.g., EU 2 µg/kg AFB_1_), new interventions can only gain traction if they demonstrate compliance across multiple regulatory regimes. This creates both practical and economic barriers: low-cost and regulatorily accepted options such as ozone are closer to deployment, while cold plasma and nanomaterial-based strategies face challenges of high equipment costs, scalability validation, and unresolved safety assessments. Similarly, biosensing platforms, though highly sensitive, require demonstration of robustness in industrial settings and regulatory recognition before integration into quality assurance systems. In general, the economic feasibility and scalability of these technologies from laboratory validation to industrial processing lines remain critical determinants of their adoption as transformative tools in pistachio safety management (PSM).

## 3. Conventional Methods for Aflatoxin Reduction

### 3.1. Thermal Treatments

Thermal processing is one of the most widely explored strategies for reducing AFs in pistachios, relying on the sensitivity of the toxin molecules to heat and moisture. The principal mechanism involves structural degradation of the difuran and lactone moieties of AFs, with hydrolytic reactions accelerated when matrix moisture is present [35,36]. Conventional roasting has been tested at temperatures between 90 and 150 °C for durations of 30 to 60 min. Results consistently show that treatments below 90 °C have little effect, while those above 150 °C compromise product color, flavor, and texture without producing proportionally greater detoxification. Roasting of contaminated pistachios at 150 °C for 30 min led to a ~60–65% reduction in AFB_1_, confirming the strong time–temperature dependence of toxin loss [3,36]. Acid-assisted roasting substantially enhances efficacy: pre-wetting pistachio kernels with lemon juice or citric acid (sometimes with added salt) and then roasting at 120 °C for 60 min achieved up to 93% reduction in AFB_1_. The synergistic effect is attributed to proton-catalyzed opening of the lactone ring combined with thermal degradation, which destabilizes the molecule far more efficiently than heat alone [37].

Alternative heating methods, such as microwave and infrared roasting, have also been studied. Both provide rapid volumetric heating, which speeds up the breakdown of AFs but depends heavily on treatment uniformity. When kernel moisture content is carefully controlled, microwave or infrared roasting can reduce AFB_1_ by 50–70%, though non-uniform heating can leave untreated “cold spots” where contamination persists [3,35]. Infrared roasting has the added benefit of improving microbial safety while preserving sensory properties better than prolonged conventional roasting [38]. Microwave heating has been reported to achieve significant reductions in a shorter time, though optimization is necessary to balance detoxification with quality [3]. Beyond pistachios, extrusion and pressure-based cooking have been benchmarked in peanuts and cereals, showing reductions of 70–80% under high-temperature, high-moisture conditions, and although not widely applied in pistachio processing, these findings demonstrate the potential of combined heat and pressure as a more aggressive strategy [13,35].

Overall, thermal treatments provide practical and scalable options for AF reduction in pistachios since they can be integrated into existing roasting lines. Their effectiveness, however, is influenced by kernel type, initial moisture, and processing parameters [3]. Complete detoxification is rarely achieved with heat alone, but combining thermal methods with acidulants or subsequent polishing steps such as ozone or adsorption can bring AF levels closer to regulatory limits while maintaining the sensory and nutritional quality of pistachios [3,36].

### 3.2. Physicochemical Treatments

#### 3.2.1. Radiation-Based Approaches

Radiation-based approaches for AF reduction involve both ionizing (i.e., γ-rays, electron beam, and X-rays) and non-ionizing radiations (e.g., ultraviolet C (UV-C)) [35]. Gamma irradiation penetrates deeply into food matrices, yet AFs are relatively resistant to direct photolysis. Their degradation occurs primarily through indirect mechanisms, where free radicals like hydroxyl (^•^OH), hydrogen (^•^H), and peroxyl (ROO^•^) formed during water radiolysis attack the terminal furan ring and lactone group of AFB_1_, producing derivatives with lower biological activity [39]. Makari et al. [40] investigated the effect of Co-60 γ-irradiation on *A. flavus*, AFB_1_, and the qualitative properties of pistachio nuts. Their results showed that γ-irradiation at 4 and 6 kGy doses diminished ~5 logs of the viable count of fungal spores where a decrease in the concentration of AFB_1_ by 73.3% and 86.4% was, respectively, recorded. Lower doses (≤2 kGy) caused minimal quality changes, while higher doses led to reductions in phenolic content, antioxidant activity, chlorophylls, and carotenoids, along with increases in lipid oxidation and darker color. They concluded that γ-irradiation above 2 kGy can effectively reduce fungal and AF contamination, but lower doses are preferable for maintaining pistachio quality [40]. Similarly, Rezaie and Zareie assessed γ-irradiation using radioactive granite as a natural source and reported that pistachio packages exposed to granite beds (2–6 kg) for 3–9 days exhibited 47–81% reductions in AFB_1_, without affecting protein and fat contents [41]. Hassanpour et al. [42] developed a simulation-assisted method for reducing AFB_1_ in pistachios using γ-irradiation from radioactive granite rock. HPLC confirmed up to 97% reduction, while Monte Carlo N-Particle eXtended (MCNPX) simulations modeled dose distribution and supported an empirical formula predicting AF reduction with 84–87% accuracy [42]. Dose-dependent reductions of about 68.8–84.6% in AFB_1_ have been reported in unpeeled/peeled pistachios [35,43]. Similar decreases were earlier observed in rice (87.8%) at 10 kGy [36], corn (66%) at 25 kGy [44], corn (69.8–94.5%) at 1–10 kGy [45], and soybean (69.8–94.5%) at 1–10 kGy [46]. However, complete elimination of AFs is rarely achieved, and greater efficiency may be obtained when irradiation is combined with other decontamination methods [47].

Electron beam irradiation (EBI), generated by linear accelerators, also relies on radical-mediated oxidation. Unlike other methods, it presents distinct advantages such as short processing times, inline operation, low heat input, and precise dosage control [48]. AFB_1_ contamination in pistachio kernels was dose-dependently reduced in a range from 38.84% at 1 kGy to 77.17% at 7 kGy. Although higher doses improved decontamination efficiency, doses above 5 kGy adversely affected organoleptic properties [49]. EBI effectively reduced *A. flavus* spores and AFB_1_ contamination in pistachios, with significant degradation observed at 4–6 kGy. Even though doses ≥ 2 kGy improved some quality traits, higher doses (>4 kGy) negatively impacted physicochemical properties, indicating that ≤2 kGy offers a balance between safety and quality [16]. Both γ-irradiation and EBI have been reported to reduce AFB_1_ levels in cereals by approximately 67–69% at 25 kGy [41]. However, studies in aqueous systems reveal multiple degradation products, some of which still retain mutagenic activity, emphasizing the need for thorough safety evaluation [50,51].

X-rays are generated by electrical machines using target materials such as gold or tantalum and have relatively low penetration ability. AFB_1_ was dose-dependently reduced by X-ray irradiation with a maximum degradation (81%) at 10 kGy. Several degradation products were also detected, and cell assays showed that treated AFB_1_ had lower toxicity than untreated AFB_1_ [52]. Moreover, UV-C irradiation degrades AFs through direct photon absorption at 222 nm, 265 nm, and especially 362 nm, which excites AFB_1_ and increases susceptibility to photolysis [53]. This process produces 10–12 identifiable degradation products via modification of the double bond in the terminal furan ring and cleavage of the lactone ring, typically following first-order kinetics [35,54]. UV-C irradiation at 265 nm significantly reduced AFs in ground nut, walnut, pistachio nut, and almond, with complete elimination of AFG_2_ after 15 min and 100% degradation of AFG_1_ in almonds and pistachios. Prolonged exposure (45 min) achieved up to 96.5% reduction in AFB_1_, and the degradation of total AFs followed first-order kinetics [55].

#### 3.2.2. Ozone Oxidation

Ozone (O_3_) has been recognized as a generally recognized as safe (GRAS) antimicrobial agent, approved by the U.S. FDA for food treatment, storage, and processing [56]. Commercially, it is generated through UV irradiation or corona electric discharge. Its strong oxidizing potential has enabled applications in water remediation, fresh produce and juice decontamination, pesticide degradation, and pest control [35,57].

Ozone initiates detoxification of AFs via electrophilic attack on the C8–C9 double bond of the terminal furan ring, causing the development of primary ozonides that subsequently rearrange or fragment into lower-toxicity products like aldehydes, ketones, and organic acids [35,58]. Akbas and Ozdemir reported that ozone treatment could partially degrade AFs in pistachio kernels and ground pistachios spiked with different AFs (i.e., B_1_, B_2_, G_1_, and G_2_). Pistachios treated with gaseous ozone at 5–9 mg/L for 140 or 420 min (20 °C, 70% RH) revealed that degradation efficiency increased with exposure dose and time. Under the most severe condition (9 mg/L for 420 min), AFB_1_ and total AFs were reduced by ~23–24% in kernels, while only ~5% decrease was achieved in ground pistachios. Interestingly, kernel ozonation did not significantly alter pH, moisture, fatty acid composition, or sensory attributes, whereas ground pistachios showed profound changes in organoleptic properties after treatment [59]. Baazeem et al., concluded that gaseous ozone (50–200 ppm for 30 min) reduced germination and populations of *A. flavus* on pistachios, but in some cases stimulated AFB_1_ production, especially at high water activity (0.98 aw), indicating that while O_3_ can suppress fungal growth, its effects on toxin synthesis are complex and dependent on environmental conditions [60]. Shakerardekani and Saberi investigated the impact of gaseous ozone on contaminated pistachios and found that treatments of 3–5 h, with intermittent stirring, significantly reduced microbial load and completely degraded detectable AFs (i.e., AFB_1_, AFB_2_, AFG_1_, AFG_2_; from 35.1 ppb total in controls to 0 ppb after 5 h). Longer ozonation times increased peroxide values, though all remained below the 1 meq/kg limit. Sensory tests indicated no adverse changes in flavor, texture, or color, except for some aroma changes after 5 h. They recommended the ozonation for 180 min with 20 min-interval stirring can be considered the optimal treatment to balance microbial and AF reduction with quality preservation [61].

A combined treatment of citric acid immersion (3 N), ozone exposure (30 min), and UV-C radiation (36 h) led to a high degradation of AFB_1_ and AFB_2_ (>90%) as well as AFG_1_ and AFG_2_ (>99%) in contaminated pistachios, without significant changes in nutritional composition or sensory quality. The combined treatment of ozone, UV-C, and citric acid showed more effectiveness than any single intervention [17]. Acidic conditions promote hydrolysis and lactone ring opening, converting AFB_1_ into less toxic derivatives such as AFB_2_. There was a synergistic effect in the concurrent use of ozone and organic acids (e.g., citric acid). Acids weaken the lactone structure, making the molecule more vulnerable to ozone attack, while ozone accelerates further oxidation and breakdown of the destabilized AF intermediates. This explains why citric acid pre-treatment followed by ozone exposure (or ozone + UV-C) results in a remarkable detoxification in pistachios compared with the effect of ozone alone [36].

#### 3.2.3. Acid/Alkaline Chemical Treatments

Acid and alkaline treatments have been applied to pistachios for AF decontamination, as acids can disrupt fungal cells and promote chemical degradation of AFs, while alkaline conditions open the lactone ring of AFs, leading to structural changes that reduce their toxicity [62,63]. The potent antifungal activity of salicylic acid against *A. flavus* in pistachios has been demonstrated. In vitro experiments showed complete growth suppression at 9 mmol/L, while in vivo assays confirmed significant inhibition, particularly in intact pistachios compared with injured ones. These findings suggest that postharvest application of salicylic acid may be an ideal treatment to reduce *A. flavus* infection and AFB_1_ production in pistachio fruits [62]. Dehydroacetic acid and ozonated water have been evaluated for their ability to control *A. flavus* growth and AFB_1_ accumulation in pistachios. Dehydroacetic acid alone and in combination with ozonated water completely inhibited fungal growth and meaningfully decreased AFB_1_ levels (1.5–1.59 µg/kg), compared with much higher contamination in control and ozonated water-only groups (~6 µg/kg). These results indicate that dehydroacetic acid possesses stronger antifungal and inhibitory activity than ozonated water, suggesting its potential application in the pistachio industry to prevent fungal contamination and AF accumulation [64]. Jubeen et al. [65] tested citric, lactic, and propionic acids as food-grade organic treatments for AF decontamination in nuts. At 9% concentration and 15 min exposure, AFs were significantly reduced in pistachios (99.9%), walnuts (~99%), and peanuts (96.1%). Citric acid was the most effective organic acid to eradicate AFs. The detoxification mechanism involved hydrolysis of the lactone ring of AFB_1_, producing less toxic derivatives such as AFD_1_ [65].

Compared to acidic conditions, fewer studies have assessed the use of alkaline treatment for AF reduction in pistachios. Vidal et al. [66] evaluated the effects of potassium hydroxide (pH 12), trifluoromethanesulfonic acid, protease, α-amylase, and cellulase on AF-contaminated maize. They found that potassium hydroxide eliminated AFs, demonstrating the strong detoxification potential of alkaline hydrolysis. Trifluoromethanesulfonic acid and protease showed no effect, while α-amylase and cellulase released only small additional amounts of bound AFs (15% and 13%). Overall, alkaline treatment was the most effective method for AF decontamination [66]. A recent study has shown that weakly alkaline conditions can effectively reduce AF contamination in foods. At pH 9, AFB_1_ and AFG_2_ were reduced by more than 50% and 95% within 24 h, respectively, whereas acidic conditions had no effect. The degradation was attributed to lactone ring opening, which lowers toxicity, and genotoxicity assays confirmed reduced harmful effects of AFB_1_. These findings show weak alkaline treatment as a promising detoxification strategy for AF-contaminated food and feed without relying on strong chemicals [63]. The consistency between strong (pH 12) and weak (pH 9) alkaline hydrolysis in maize reveals the vital role of lactone ring instability in AF degradation. Although strong bases ensure complete breakdown of AF structures, even mild alkalinity can trigger significant reductions in AF toxicity via partial hydrolysis. This evidence demonstrates that alkaline treatment may be one of the most reliable chemical strategies for AF decontamination in pistachios.

## 4. Emerging Nonthermal Methods for Aflatoxin Reduction

### 4.1. Photon/Energy-Based Methods

#### 4.1.1. Pulsed Light

Pulsed light (PL) is an FDA-approved nonthermal technology that may act as an alternative to, or complement for, conventional decontamination methods. PL generates short, high-intensity flashes of broad-spectrum white light, which includes UV, visible, and infrared regions. The synergy of these spectra rapidly disrupts microbial cell walls and nucleic acids within seconds [67]. In excess of microbial inactivation, PL has gained attention for its ability to degrade mycotoxins. Moreau et al., reported that eight flashes of PL degraded zearalenone, deoxynivalenol, AFB_1_, and ochratoxin by 85%, 72%, 93%, and 98% in solution, respectively [68]. For AFs, PL acts by inducing photolysis of AFB_1_ through UV absorption near 362 nm, leading to lactone ring cleavage and bond modification. Wang et al., reported a significant reduction in AFB_1_ in water (92%) and rice bran (~90%) within seconds, without detectable toxicity of degradation products [67]. However, further validation in nuts such as pistachios is still needed to optimize treatment conditions and ensure the safety of degradation products. Zhou et al. [69] have recently assessed the use of PL against *A. flavus* and AF contamination in peanuts. They showed that PL inactivated fungal spores, suppressed AFB_1_ production by downregulating biosynthetic genes, and disrupted cell membrane, antioxidant, and energy metabolism systems. At 13.5 J/cm^2^, PL degraded AFB_1_ and AFG_1_ by 61.6% and 48.0%, respectively, likely by cleaving the C8–C9 double bond. PL as a promising postharvest method can be applied to control AF contamination in other agro-products [69]. Although pistachio-specific studies are not yet available, evidence from similar matrices proves PL as a promising strategy that warrants further investigation for pistachio decontamination.

#### 4.1.2. Cold Plasma

CAP technology has attracted growing interest in the food sector over the past decade due to its potential for safe and sustainable processing. Plasma is an ionized gas with charged particles and reactive species that can be generated under electromagnetic fields. With advances in plasma sources, cold plasma can now be applied at atmospheric pressure, making it suitable for food applications. The reactive species produced during treatment can inactivate microorganisms, degrade toxins, and induce beneficial chemical changes, which has encouraged its exploration for AF reduction in nuts such as pistachios. Cold plasma treatment detoxifies AFs either by blocking their formation at the fungal source or by degrading the final toxin molecules, thereby converting them into nontoxic or less-toxic products [70]. Dielectric barrier discharge (DBD) and atmospheric pressure capacitively coupled plasma (AP-CCP) are the most common CAP systems. DBD uses dielectric-covered electrodes to stabilize the discharge, while AP-CCP sustains plasma through capacitive coupling between electrodes, indicating broader and more uniform treatment [15].

Laika et al. [71] tested a surface DBD-CAP system capable of operating under ozone and nitrogen oxide regimes to degrade AFs (i.e., B_1_, B_2_, G_1_, and G_2_) and ochratoxin A. The ozone regime of CAP was especially effective, reducing AFB_1_ and AFG_1_ by up to 99%, AFB_2_ and AFG_2_ by ~60%, and ochratoxin A by ~70% after 60 min at 4 cm from the plasma source. In spiked pistachio kernels, the effect was lower because of matrix interference, but ochratoxin A still decreased by ~23% [71]. Makari et al., also applied a DBD-CAP device to analyze its significant potential for pistachio decontamination. Increasing treatment duration (up to 180 s) reduced *A. flavus* spores by 4 logs and lowered AFB_1_ levels by ~65% on glass slides and ~52% in pistachio kernels. Plasma exposure caused some quality changes, including reduced chlorophylls, carotenoids, color brightness, and protein solubility, whereas malondialdehyde levels increased. However, total phenolic content was unaffected and antioxidant activity slightly improved. Accordingly, they concluded that this system can effectively decrease fungal growth and AF contamination with fewer adverse quality effects than conventional decontamination methods, supporting its use as a promising nonthermal strategy for pistachio safety [72]. A recent study investigated CAP for reducing *A. flavus* and AF contamination in pistachios while monitoring quality traits. The most effective conditions (20 kV, 15 min, and argon–air mixtures) resulted in a 5.14 log reduction in *A. flavus* and maximal AF reductions of 83.7% (AFB_1_), 74.4% (AFB_2_), 41.4% (AFG_1_), and 50.7% (AFG_2_). Furthermore, pistachio quality parameters showed no significant differences compared to untreated controls, confirming CAP as a promising decontamination method with minimal quality loss [6]. Bi and Ma have recently reported that DBD-CAP treatment effectively degraded AFB_1_ in pistachios with maintaining most nutritional qualities. This treatment caused no significant changes in protein, acid value, or peroxide value, but increased γ-aminobutyric acid content by more than twofold and reduced flavonoid levels. It also improved the ω-6/ω-3 fatty acid ratio toward the recommended value and extended shelf life to 21–30 days depending on the quality parameter measured. They found that this nonthermal technology not only detoxified pistachios from AFB_1_ but also contributed to improved preservation and nutritional quality [73]. Bakhtiyari-Ramezani et al., also investigated DBD plasma on pistachios and confirmed its ability to detoxify AFs [74]. Devi et al., had earlier shown that DBD plasma operating at 60 W reduced more than 95% of AFs on pistachios [75]. However, Bakhtiyari-Ramezani et al., reported that treatment time is critical, so that short exposures (≤10 min) preserved quality, whereas prolonged treatments (20–40 min) risked nutritional degradation [74].

**Figure 2:**
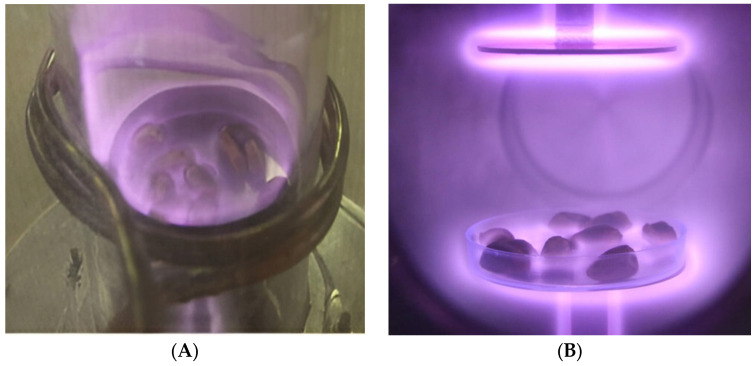
ICP (**A**) and DC-DP (**B**) devices to treat *A. flavus* from the surface of pistachio nuts (reprinted with permission from Ghorashi et al. [77]. Copyright© (2020) Springer Nature).

An AP-CCP device using argon gas was applied to reduce *A. flavus* contamination of pistachios. The system, designed for mobility and large-scale treatment, led to a 4-log (66.6%) reduction in the fungus under conditions of 100 W, atmospheric pressure, and 10 min irradiation, representing AP-CCP as a practical approach for controlling fungal pathogens in pistachios [76]. Ghorashi et al. [77] evaluated three cold plasma devices, including AP-CCP, inductively coupled plasma (ICP; Figure 2), and direct current diode plasma (DC-DP; Figure 2), for inactivation of *A. flavus* on pistachio nuts. Their results showed that the AP-CCP device could reduce the fungal count by 4-log at 100 W for 10 min under atmospheric pressure, whereas the DC-DP device obtained the maximum fungi reduction of 5 logs at 300 W for 20 min under 1 Torr vacuum pressure. Although DC-DP was more effective in terms of microbial inactivation, AP-CCP was considered the optimum device due to its feasibility at atmospheric pressure, cost-effectiveness, and minimal impact on nut appearance, making it more suitable for large-scale applications [77].

A study on fresh pistachios evaluated the combined effect of chitosan edible coating (0.5–1.5%) and cold plasma treatment (60–120 s) during 180 days of storage. The combination of 1.5% chitosan and 120 s plasma preserved hardness and color, minimized peroxide values, mold and yeast counts, and significantly reduced AF levels, without altering sensory quality [70]. The combined application of UV irradiation and CAP was more effective than either treatment alone in decontaminating aflatoxigenic fungi in dried pistachios. Simultaneous UV-CAP treatment at 80 W for 15 min led to the highest fungal reduction (3.7 CFU/g) without significant changes in pH or antioxidant activity [78].

### 4.2. Adsorptive/Nanomaterial-Based Methods

Adsorptive and nanomaterial-based approaches have gained increasing attention as effective strategies for AF control in pistachios. These methods rely on the physical binding of toxins to microbial cell wall components, biopolymers, or engineered nanostructures, reducing toxin bioavailability without altering product quality [79]. Recent advances, including bio-based adsorbents, functionalized nanoparticles, and active packaging films, demonstrate their potential as sustainable and scalable solutions for pistachio safety.

Rahaie et al. [80] earlier assessed the potential of *Saccharomyces cerevisiae* to bind AFs in pistachios, with surface binding efficiencies of 40% and 70% at initial concentrations of 10 and 20 ppb, respectively, during the exponential phase. Acid and heat treatments enhanced this ability, reaching up to 73% and 75%, respectively. Binding was rapid, saturating within 2–3 h, and appeared to be a physical process. The yeast immobilization did not affect pistachio quality parameters such as color, texture, or peroxide value. Both viable and nonviable cells proved effective, supporting *S. cerevisiae* as a promising bio-based approach for AF reduction in pistachios [80]. Abdolshahi et al. [81,82] reported that edible coatings enriched with *S. cerevisiae* cell wall mannoproteins significantly inhibited *A. flavus* growth and reduced AF levels in pistachios. The coating delayed mycelial development and sporulation, while AFB_1_ was reduced by ~42.8% at a mannoprotein concentration of 1.5%. They mentioned that yeast-derived mannoproteins not only limit fungal growth but also bind AFs, supporting their potential as natural bioactive agents for AF control in pistachios [81]. These researchers earlier pointed out that mannoprotein extracted from the cell wall of *S. cerevisiae* can bind AFs in contaminated pistachios. Soaking pistachios in mannan solution reduced AFB_1_ by up to 84%, with most binding occurring within the first 5 min of contact. Incorporating mannan into a gelatin-based coating also proved effective, lowering AFB_1_ levels by ~83%. These results confirm that yeast-derived mannoprotein, applied directly or as part of edible coatings, can be considered a potential adsorptive approach for reducing AF contamination in pistachios [82].

**Figure 3:**
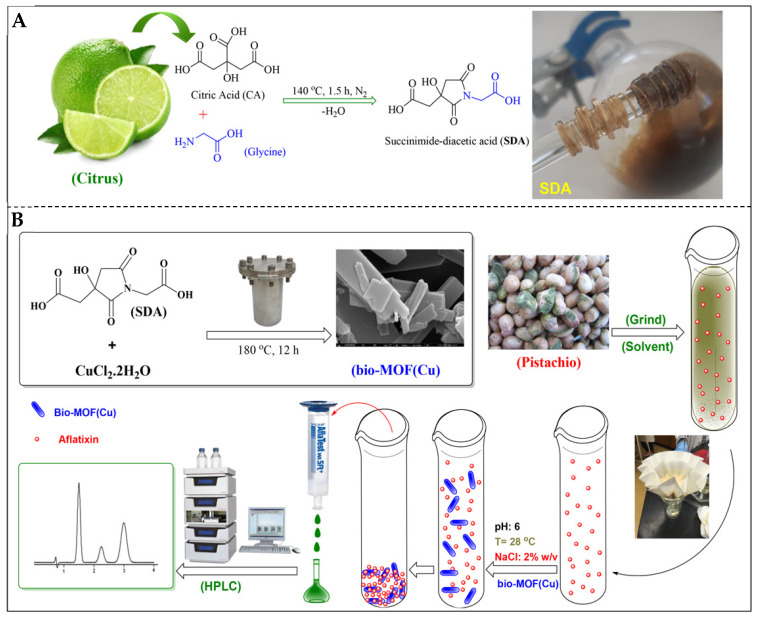
(**A**) The preparation of SDA. (**B**) Bio-MOF(Cu) prepared by hydrothermal synthesis for AF adsorption (reprinted with permission from He et al. [1]. Copyright© (2025) Elsevier).

He et al. [1] developed an eco-friendly bio-MOF(Cu) synthesized from a novel cyclic succinimide diacid (SDA, 95%) linker (condensation of citric acid and glycine; Figure 3A) and CuCl_2_·2H_2_O for AF removal in pistachios. The hydrothermally synthesized bio-MOF(Cu) was subsequently applied to pistachio extracts spiked with AFs, where it rapidly captured toxin molecules through surface interactions, and the remaining solution was filtered before HPLC analysis. The schematic workflow (Figure 3B) illustrates the synthesis, adsorption process at pH 6 with 2% NaCl, and subsequent chromatographic detection, confirming the high removal efficiency of the nanomaterial. The bio-MOF achieved rapid adsorption efficiencies of >98.6% for AFB_1_ and 96.9% for AFB_2_ within 10 min under optimized conditions (pH 6, ambient temperature, 2% NaCl, 5 mg/10 mL solution). Adsorption followed the Langmuir isotherm model, with maximum capacities of 10.65 mg/g for AFB_1_ and 11.83 mg/g for AFB_2_, indicating monolayer adsorption on a homogeneous surface. The material retained ~78% efficiency after five reuse cycles, demonstrating high recyclability. This study evinces bio-MOFs as promising nanostructured adsorbents for pistachio decontamination, showing efficiency, sustainability, and potential scalability [1].

Karami-Osboo et al. [83] developed a polydopamine-coated magnetic *Spirulina* nanocomposite for adsorptive removal of AFs in pistachios. The nanomaterial, characterized by mesoporous structure and strong magnetic properties, efficiently extracted AFB_1_, AFB_2_, AFG_1_, and AFG_2_ via hydrogen bonding, hydrophobic, and π–π interactions. Optimal conditions enabled rapid adsorption equilibrium within 1 min, with recoveries of 72–95% and low LODs (0.02–0.07 ng/g). The method was validated using spiked samples and certified reference material, showing accuracy comparable to immunoaffinity column clean-up. These findings demonstrate the potential of polydopamine-modified magnetic *Spirulina* as an eco-friendly and effective adsorbent for routine monitoring and decontamination of AFs in pistachios [83].

Ansari et al. [84] showed that kefir grains could reduce AFB_1_ in pistachio paste by up to 96% under optimized conditions (20 ng/g toxin, 20% kefir grains, 6 h, 30 °C). Both viable and non-viable grains were effective, indicating that the mechanism was mainly surface binding rather than metabolic degradation [84]. Rahaie et al. [85] compared surface binding abilities of *S. cerevisiae* (40–70%) and *Lactobacillus rhamnosus* GG (35–60%) to pistachios for reducing AF contamination at initial concentrations of 10 and 20 ppb. Acid treatment markedly enhanced binding, reaching up to 73% for yeast and 90% for *L. rhamnosus*, while heat treatment also improved efficiencies (55–75% for yeast, 85–90% for *L. rhamnosus*). They also showed that microbial immobilization did not affect pistachio quality parameters such as color, texture, or peroxide value [85]. Lactic acid bacteria primarily reduce AFs by binding them to cell wall components, including polysaccharides, peptidoglycans, and proteins. Acid and heat treatments disrupt the cell wall, exposing additional binding sites and enhancing toxin attachment. This process is physical rather than metabolic, and both viable and non-viable cells can sequester AFs, thereby lowering their bioavailability [86].

Nanotechnology-based food packaging provides improved barrier and antimicrobial properties that can control fungal growth and toxin production while extending the shelf life of nuts. Such active packaging systems are particularly valuable for pistachios, which are highly susceptible to AF contamination during storage [87,88]. Nanocomposite packaging films enriched with silver nanoparticles and quinoa peptides were developed to reduce AF contamination in pistachios. Pistachios artificially inoculated with *A. flavus* and stored at 25 °C for 30 days showed significant reductions in AFB_1_ and total AFs when packaged in films containing both agents, with the strongest effect observed at 35% incorporation. The synergistic action of silver nanoparticles and quinoa peptides successfully suppressed fungal growth and toxin accumulation while extending pistachio shelf life. These results show their potential as active nanomaterial-based packaging solutions [89].

### 4.3. Bioactive/Biological-Based Methods

#### 4.3.1. Natural Bioactive Compounds

Natural bioactive compounds are considered eco-friendly alternatives to control fungal growth and AF contamination in pistachios. Postharvest application of melatonin (1000 µM) to pistachios reduced *A. flavus* decay and AFB_1_ accumulation during storage at 25 °C for 18 days. The treatment inhibited spore germination and germ tube elongation, lowered oxidative stress indicators (H_2_O_2_ and malondialdehyde), and suppressed lipoxygenase activity, thereby preserving unsaturated fatty acids. In addition, melatonin enhanced phenylalanine ammonia-lyase activity, increased phenol and flavonoid content, and improved antioxidant capacity, indicating activation of the phenylpropanoid pathway. These effects not only limited fungal infection and toxin production but also helped maintain the nutritional quality of pistachio kernels [90]. Similarly, exogenous β-aminobutyric acid treatment at 7.5 mM suppressed *A. flavus* spore germination and germ tube elongation in pistachios, reduced decay and AFB_1_ accumulation during storage, and enhanced phenol and flavonoid levels, antioxidant capacity, and phenylalanine ammonia-lyase activity, supporting the role of phenylpropanoid pathway activation in both fungal resistance and quality preservation [91].

Plant-derived bioactive compounds and essential oils have strong antimicrobial and anti-mycotoxigenic properties, but their volatility and instability often limit direct application. Encapsulation improves their stability, controlled release, and efficacy, making them promising natural agents for controlling fungal growth and AF contamination. Khodaverdi et al. [92] investigated a neem seed extract formulation containing azadirachtin on *A. flavus* and AF production in pistachios. The extract, formulated with Tween 20 and sunflower oil, slightly increased fungal growth but reduced AFB_1_ by ~17% and B_2_ by ~23% in culture. Similar reductions were observed on pistachios, although fungal growth was not inhibited. The effect was mainly attributed to azadirachtin, which, besides its insect-repellent role, contributed to lowering AF accumulation. This study suggests neem extract can help reduce pistachio contamination by combining insect control with partial suppression of AF biosynthesis [92].

Shahdadi et al. [93] have recently analyzed the chemical composition and antimicrobial potential of essential oil extracted from pistachio hulls containing α-pinene (47.36%), terpinolene (10.57%), and limonene (9.13%). In antifungal assays, pistachio hull essential oil showed strong inhibitory activity against *A. flavus*, with efficacy comparable to fluconazole, and also suppressed *A. parasiticus*. The fungicidal effect (minimum fungicidal concentration/minimum inhibitory concentration (MFC/MIC) ratio ≤ 4) indicates that pistachio hull essential oil can directly inhibit fungal growth [93]. Moreover, the hull contains hydrolyzable tannins and phenolic compounds (e.g., gallic acid and quercetin) previously reported to suppress AFB_1_ biosynthesis [94,95]. Gallic acid acts by altering intracellular pH and disrupting energy metabolism, while quercetin interferes with membrane potential and increases membrane permeability, thus impairing fungal growth and toxin production [93]. The antifungal and anti-aflatoxigenic effects of *Zataria multiflora* essential oil encapsulated in chitosan nanoparticles (293 nm) with high encapsulation efficiency (87.4%) on pistachios were investigated. Both free and encapsulated *Z. multiflora* essential oil prevented *A. flavus* growth (95%) and reduced AFB_1_ production (92%) in liquid media. When applied to contaminated pistachios, *Z. multiflora* essential oil and its nanoencapsulated form with CS chitosan decreased AFB_1_ levels by 90.5% and 99%, respectively. These findings suggest that nanoencapsulation of this bioactive-rich essential oil is a potent natural antifungal agent for controlling AF contamination in pistachio kernels [96].

#### 4.3.2. Microbial Biocontrol Agents

Microbial biocontrol represents an eco-friendly and sustainable approach to managing AF contamination in pistachios. Various bacteria, yeasts, and probiotics have shown the potential in limiting fungal growth, reducing sporulation, and transforming or adsorbing AFs, making them promising alternatives to chemical treatments.

Siahmoshteh et al. [97] demonstrated that *Bacillus amyloliquefaciens* (UTB2) and *B. subtilis* (UTB3), isolated from pistachio orchard soils, effectively inhibited *A. parasiticus* growth and AF production. Both strains significantly reduced fungal mycelial growth in vitro, likely through the release of antimicrobial lipopeptides, and degraded AFs enzymatically rather than by adsorption, with total reductions of ~80–84% after 120 h in liquid culture. Among four pistachio cultivars tested, ‘Ahmad-Aghaei’ was most susceptible to infection and was selected for further assays. In vivo, co-inoculation with *Bacillus* strains decreased fungal incidence and spore counts, while *B. subtilis* resulted in a greater log reduction. Maximum AF reduction on pistachios occurred at 8 days post inoculation (dpi) (52.4% for UTB2 and 45.9% for UTB3), although this effect diminished by 12 dpi due to decreased bacterial activity. Overall, both *Bacillus* strains showed promising antifungal and anti-mycotoxigenic potential, particularly under in vitro conditions, indicating their suitability as microbial biocontrol agents for pistachio AF management [97]. Farzaneh et al. [98,99] reported that *B. subtilis* UTBSP1 capably inhibited *A. flavus* growth and AFB_1_ contamination in pistachios. Treatments with higher bacterial concentrations (10^7^ CFU/mL) almost entirely suppressed fungal colonization and eliminated detectable AFB_1_. The cell-free supernatant of UTBSP1 disrupted spore viability, and bioactive fractions were identified as lipopeptides from the surfactin and fengycin families. These compounds acted synergistically, providing strong antifungal activity [98]. In another study, these researchers mentioned that this strain significantly reduced AFB_1_ in nutrient broth (85.7%) and contaminated pistachio kernels (up to 95%) within 5 days. The cell-free supernatant also achieved 78% degradation, with optimal enzymatic activity at 35–40 °C, confirming that extracellular enzymes were responsible. The degraded product lost fluorescence, indicating structural alteration of AFB_1_ and supporting its detoxification potential [99]. Afsharmanesh et al. [100] investigated γ-irradiation mutagenesis of *B. subtilis* UTB1, to enhance its biocontrol activity against *A. flavus* R5. Among 500 colonies screened after exposure to 0.1–3 kGy, 45 colonies showed improved antifungal activity, and eight mutants were selected based on polymorphism patterns. Six mutants produced significantly higher levels of lipopeptides (e.g., surfactins, fengycins, and iturins), with bioautography linking fungal inhibition primarily to iturin production. In pistachio nut assays, mutants M419 and M464 markedly suppressed *A. flavus* sporulation and reduced AFB_1_ accumulation compared to the wild-type strain. These results demonstrate γ-irradiated *B. subtilis* mutants as new microbial biocontrol agents for managing AF contamination in pistachios [100].

Moradi et al. [101] evaluated 13 native yeast strains isolated from pistachio orchards in Iran for their antagonistic activity against *A. flavus* and AFB_1_ production. In vitro tests showed that yeasts inhibited *A. flavus* mycelial growth by 32–61%, depending on the assay, and reduced AFB_1_ levels by 90.6–98.3%. Five promising strains, identified as *Pichia kudriavzevii* and *Lachancea thermotolerans*, were further tested on soil, pistachio hulls, and leaves, where they significantly reduced *A. flavus* colony-forming units by up to 99%. These findings show native yeasts as effective microbial biocontrol agents with strong potential for reducing AF contamination in pistachio orchards [101]. Pakizeh et al., showed that adding *Bifidobacterium lactis* to pistachio paste with improving sensory acceptance reduced AFB_1_ contamination by up to 73% under laboratory conditions, with an optimal reduction of 59.4% at 25 °C for 26 days (10^9^ CFU/g) [102].

### 4.4. Comparative Assessment of Nonthermal Decontamination Methods

A comparative summary of the efficacy, quality impact, scalability, technological readiness, and cost considerations of nonthermal decontamination methods is presented in Table 1. PL rapidly photodegrades AFs via UV-driven bond cleavage and has delivered high removal in model systems. In nuts, matrix/geometry effects constrain efficacy. A recent peanut work showed 61.6% AFB_1_ and 48.0% AFG_1_ degradation at 13.5 J cm^−2^, while UV-based optical treatments on pistachios achieved 22% AFB_1_ reduction at 3 h and 58% at 13 h under 87.5 µW cm^−2^ [15], illustrating that dose delivery, shadowing, and penetration limit outcomes on irregular kernels. Ozone is well-documented across nuts and pistachios; AFB_1_/AFG_1_ are more ozone-sensitive than AFB_2_/AFG_2_ [36], but excessive exposure can affect lipid stability or sensory attributes, so process windows must balance detoxification with quality [15]. By contrast, CAP generates reactive gas species that simultaneously inactivate *Aspergillus* and degrade AFs with minimal heating and no chemical residues; nut studies show ~4–5 log reductions in *A. flavus*/*parasiticus* in minutes (e.g., 5 min at 655 W with dry air on hazelnuts) [103], and pistachio-focused CAP work reports near-complete reductions for AFB_1_/AFG_1_ (up to ~99%) under ozone-rich regimes on solid substrates, though matrix interference lowers effects in spiked kernels (e.g., ~23% for ochratoxin A) and underscores the need for dose uniformity and scale-up studies. Nanomaterial-based adsorption/packaging presents a complementary, non-destructive route by selectively binding AFs (e.g., polydopamine-coated magnetic sorbents; silver-nanoparticle/peptide films trialed on pistachio) and can be integrated into coatings or postharvest contact steps, but questions remain on regeneration, migration, and regulatory acceptance for industrial lines [104].

Considering detoxification efficiency, treatment time/intensity, product quality, scalability/energy, and compliance, no single technology is universally superior. CAP currently represents the strongest speed–efficacy–quality triad for kernel-level decontamination with residue-free chemistry, yet requires standardized equipment, dose mapping on heterogeneous loads, and validated end-product toxicology at scale [11,103]. PL/UV are attractive for surface-dominant contamination and conveyor integration but need line-of-sight engineering to reduce shadowing and ensure consistent fluence on in-shell/irregular kernels [15]. Ozone is effective and already familiar to industry, but must be tuned to avoid oxidation-driven quality losses in high-lipid matrices like pistachio [18]. Recent studies explain workable windows and quality monitoring needs of pistachio. Nanomaterial adsorption is promising for selective removal/packaging-based mitigation and could pair with other hurdles, though industrial application depends on migration limits and cost-effective regeneration [104]. Overall, for real-world pistachio lines, CAP (as an in-line, short-contact, residue-free step) and carefully engineered PL/UV (for surface loads and pre-sorting) appear most ready when integrated with on-line quality controls; ozone remains a viable option with strict process control; nanomaterials are emerging as adjuncts rather than stand-alone decontaminants at present. This comparison aligns with wider nut-processing reviews emphasizing that nonthermal methods preserve sensory/nutritional attributes better than thermal steps, yet demand robust process control to ensure completeness of decontamination and product quality at scale [11,15,104].

## 5. Advanced Biosensing Platforms for Aflatoxin Monitoring

Thin layer chromatography (TLC), ELISA, and HPLC with fluorescence detection (HPLC-FLD) or coupled with mass spectrometry (HPLC-MS) are considered common analytical methods to detect mycotoxins at trace levels. Although HPLC-based techniques are highly accurate, they require trained personnel, complex sample preparation, costly equipment, and long analysis times. As a result, these methods are less practical for on-site testing and for use in regions with limited infrastructure and resources [105,106,107]. These chromatography and immunoassay techniques usually examine only a portion of samples, which cannot fully capture contamination across an entire batch, and untested or questionable samples may lead to secondary contamination. These limitations emphasize the need for rapid, nondestructive, and automated testing methods to certify the safety of nuts like pistachios [9,108].

In recent years, biosensors and sensor-based platforms have gained increasing attention as rapid, sensitive, and cost-effective alternatives for AF detection in pistachios. Unlike conventional chromatography or immunoassay methods, biosensors suggest portability, shorter analysis time, and minimal sample preparation, making them highly suitable for on-site screening and food safety assurance [109,110,111]. Different formats have been successfully applied to pistachios, including electrochemical immunosensors, aptamer-based electrochemical sensors (aptasensors), optical fluorescence and HSI systems, and other imaging tools. Each of these approaches provides distinct advantages in terms of sensitivity, selectivity, and potential integration into automated and nondestructive quality control workflows. The following subsections summarize these biosensor categories and explain their specific applications to pistachio nuts.

### 5.1. Electrochemical Immunosensors

Electrochemical immunosensors are highly important for AF determination in pistachios because they combine the specificity of antibody–antigen recognition with the sensitivity of electrochemical signal transduction, facilitating rapid and authentic detection even at trace levels. Their mechanism relies on immobilized antibodies (or antigens) that selectively bind AFs, where the resulting binding event alters the electrical properties (current, potential, or impedance) at the sensor’s electrode surface, producing a measurable signal proportional to toxin concentration [111,112,113].

**Figure 4:**
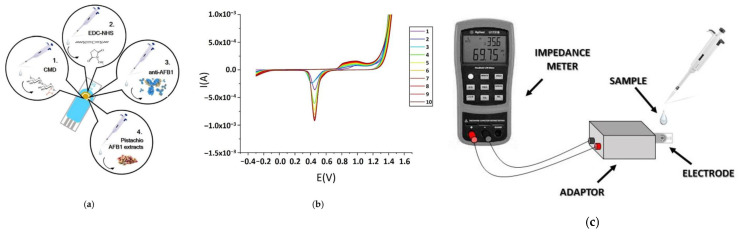
(**a**) Schematic illustration of the stepwise immobilization of anti-AFB_1_ antibodies on the gold SPE surface: (1) modification with CMD; (2) activation of carboxyl groups using EDC/NHS (1-ethyl-3-(3-dimethylaminopropyl) carbodiimide/N-hydroxysuccinimide) coupling agents; (3) immobilization of anti-AFB_1_ antibodies; (4) exposure to pistachio AFB_1_ extracts. (**b**) Cyclic voltammograms recorded during successive electrode modification steps, showing distinct electrochemical responses corresponding to each stage of functionalization and binding. (**c**) Assembly of the impedance-based biosensor. Reprinted from Kaminiaris et al. [20], under Creative Commons Attribution (CC BY) license http://creativecommons.org/licenses/by/4.0/ (accessed on 2 September 2025).

Kaminiaris et al. [20] investigated an impedimetric electrochemical immunosensor for the detection of AFB_1_ in pistachio matrices. Gold screen-printed electrodes (SPEs) functionalized with a carbo-methyldextran (CMD) layer and immobilized with monoclonal antibodies, the sensor could monitor antibody–antigen interactions via impedance spectroscopy (Figure 4). The results showed that the device resulted in an LOD of 0.5 ng/mL in standard solutions and 1 ng/mL in spiked pistachio extracts, with a reliable linear range between 1 and 100 ng/mL. Application to naturally contaminated pistachio samples demonstrated successful quantification of AFB_1_ up to ~345 ng/mL, although higher concentrations exceeded the sensor’s upper detection capability. The designed immunosensor shows an acceptable selectivity by discriminating AFB_1_ from other mycotoxins such as ochratoxin A and AFM_1_ [20].

**Figure 5:**
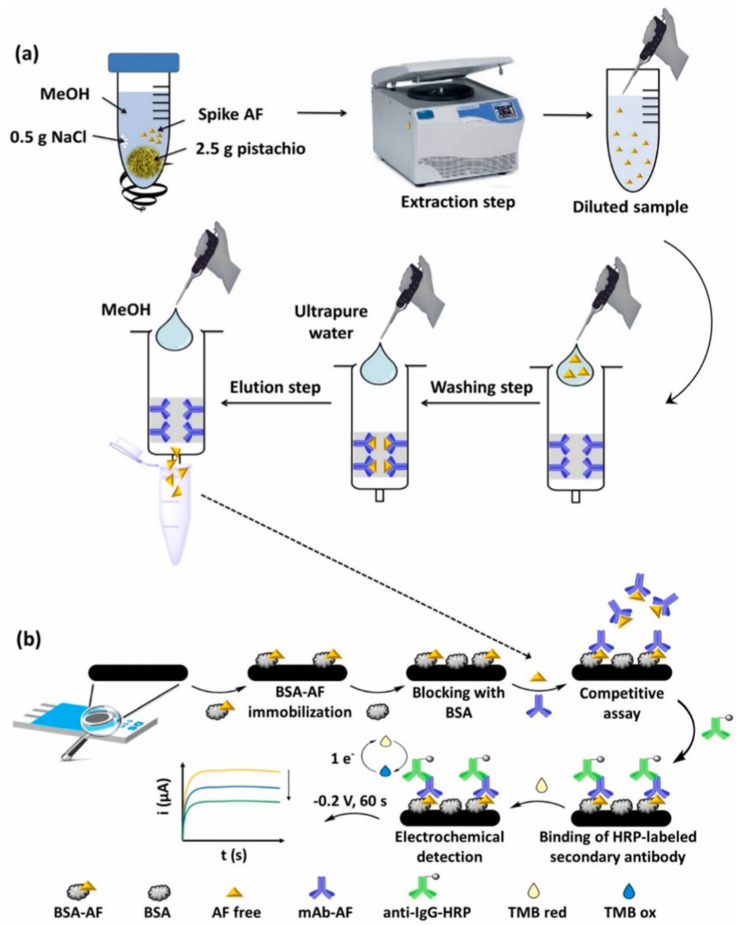
Workflow for total AF detection in pistachios. (**a**) Sample extraction: 2.5 g pistachio spiked with AFs is mixed with NaCl and methanol (MeOH), centrifuged, diluted, and purified by IAC via washing and elution steps. (**b**) Competitive electrochemical immunoassay on SPCEs: BSA–AF is immobilized and blocked with BSA; free AFs compete with immobilized conjugates for monoclonal antibodies, followed by binding of HRP-labeled secondary antibodies. TMB (3,3′,5,5′-Tetramethylbenzidine) oxidation and its electrochemical reduction at –0.2 V for 60 s generate a current signal proportional to AF concentration. Reprinted from Pérez-Fernández et al. [7], under Creative Commons CC-BY license.

Kaur et al. [22] have recently developed a highly sensitive electrochemical immunosensor for detecting AFB_1_ using a nanocomposite of amino-functionalized molybdenum-based MOF (NH_2_–Mo-MOF) and Ti_3_C_2_ MXene, deposited on SPEs. Anti-AFB_1_ antibodies were covalently immobilized on the composite surface, and electrochemical impedance spectroscopy (EIS) was used for detection. The biosensor could detect AFB_1_ in an ultra-low LOD of 8 pg/mL with a broad linear range of 0.06–50 ng/mL, demonstrating excellent reproducibility, selectivity, stability, and rapid response. The sensor was validated on spiked commercial pistachio samples via a matrix addition method. Recovery values ranged between ~97–111%, and results were in close agreement with HPLC and LC–MS analyses. The biosensor maintained its functionality even in the presence of interfering mycotoxins (e.g., zearalenone), bacterial contaminants (i.e., *Escherichia coli* and *Staphylococcus aureus*), and heavy metals (i.e., Hg^2+^ and Pb^2+^). Stability tests also showed reliable performance for up to 12 weeks under refrigerated storage. Overall, MOF/MXene composites could present synergistic properties, such as high surface area, conductivity, and effective antibody immobilization, making this biosensor a powerful and cheap platform for routine monitoring of AF contamination in pistachios [22]. A competitive electrochemical immunosensor to detect total AFs (AFB_1_, AFB_2_, AFG_1_, and AFG_2_) in pistachio was developed. The sensor was built on screen-printed carbon electrodes (SPCEs) and used a bovine serum albumin (BSA)–AF conjugate immobilized on the electrode surface, competing with free AFs in the sample for monoclonal antibodies. Detection was carried out through an enzymatic reaction with horseradish peroxidase (HRP) and chronoamperometric measurement. The optimized system showed a linear range of 0.01–2 µg/L with an LOD of 0.017 µg/L in buffer and 0.066 µg/kg in pistachio matrix. The immunosensor demonstrated good reproducibility (relative standard deviation (RSD) ~2%), stability for at least 30 days at room temperature, and high selectivity, with no significant interference from zearalenone, ochratoxin A, glucose, Na^+^, or K^+^. The method included immunoaffinity column (IAC) extraction of AFs from pistachio samples, followed by analysis with the immunosensor (Figure 5). Recovery values ranged from 87 to 106% and the cross-validated results against LC–MS/MS confirmed the constructed biosensor reliability [7].

### 5.2. Aptamer-Based Electrochemical Sensors

**Figure 6:**
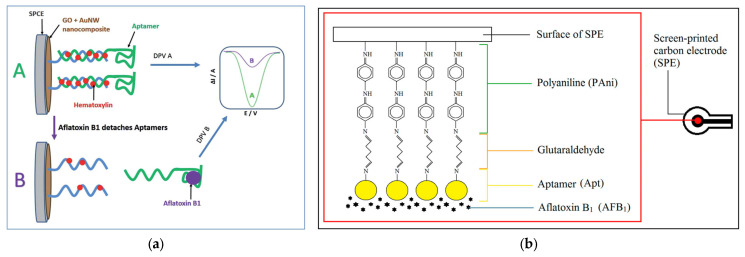
(**a**) Representation of the aptasensor working mechanism for AFB_1_ detection. (**A**) In the absence of toxin, the aptamer remains immobilized on the GO/AuNW-modified SPCE, and hematoxylin produces a strong DPV signal. (**B**) Upon AFB_1_ binding, the aptamer detaches from the electrode surface, leading to a reduced DPV signal proportional to toxin concentration (reprinted with permission from Mousavi Nodoushan et al. [114]. Copyright© (2019) Royal Society of Chemistry). (**b**) Schematic diagram of the PAni-based impedimetric aptasensor for AFB_1_ detection (reprinted with permission from Ong et al. [115], under Creative Commons Attribution (CC BY) license (http://creativecommons.org/licenses/by/4.0/ (accessed on 3 September 2025).

Aptasensors use short DNA or RNA sequences that bind specifically to AFs, converting this recognition into an electrical signal for fast and sensitive detection in pistachios. Mousavi Nodoushan et al. [114] developed a graphene oxide (GO) and gold nanowire (AuNW)-based electrochemical aptasensor for detecting AFB_1_. The design used a thiol-modified DNA probe immobilized on the GO/AuNW-modified SPCE, which hybridized with the AFB_1_-specific aptamer. In the presence of AFB_1_, the aptamer preferentially bound the toxin and detached from the electrode surface, leading to measurable decreases in differential pulse voltammetry (DPV) signals using hematoxylin as the electroactive label (Figure 6a). The sensitivity of aptasensor was affirmed with an ultra-low LOD of 1.4 pM within a linear range of 5–750 pM. Specificity studies revealed selective recognition of AFB_1_ compared to structurally similar AFs (i.e., AFB_2_, AFG_1_, and AFG_2_) and other non-specific molecules, while a good reproducibility (RSD ~5.2%) was also recorded. This aptasensor was assessed on spiked pistachio nuts at different contamination levels (i.e., 5, 50, and 200 ppb) and on naturally contaminated pistachio samples. Recoveries were close to 100%, and results correlated strongly with HPLC analysis, supporting its suitability for real-world application [114]. In general, integrating GO and AuNWs can lead to a high-surface-area, conductive nanocomposite, which can improve aptamer immobilization and signal transduction, making it a high-sensitive sensing platform to detect AFB_1_ in pistachios.

Ong et al. [115] also developed an impedimetric aptasensor to detect AFB_1_ using a polyaniline (PAni) film as a conductive support on SPCEs. A specific DNA aptamer for AFB_1_ was immobilized on the PAni layer via glutaraldehyde crosslinking, and EIS was employed to measure binding events (Figure 6b). The aptasensor demonstrated a linear detection range of 0.03–0.08 nM, with a very low LOD of 0.01 nM, and high reproducibility (RSD < 5%). The strong selectivity of the sensor for AFB_1_ was confirmed, with only partial responses to structurally similar mycotoxins (e.g., AFB_2_ and ochratoxin A). This sensor not only maintained stability for up to five days under refrigerated storage but also was regenerable, showing consistent performance over four detection cycles. Furthermore, the aptasensor was validated in real food matrices, including commercial pistachio nuts, as well as cinnamon, clove, corn, and soybean. Recovery values for pistachio were between 87.9 and 94.7%, confirming its reliability for practical application. With a rapid response time of 30 min and cost-effective fabrication, this PAni-based aptasensor provides an intriguing tool for sensitive and portable AFB_1_ detection in pistachios [115].

### 5.3. Laser-Induced Fluorescence Sensors

Laser-induced fluorescence (LIF) spectroscopy has emerged as one of the most promising optical techniques for the rapid, non-destructive detection of AF contamination in pistachios. LIF contributes to sensitive in-line or on-line screening of contaminated kernels with high precision by using the characteristic fluorescence signatures of AFs under specific excitation wavelengths [8,116,117,118,119]. Jamali Paghaleh et al. [116] introduced an in-line LIF spectroscopy method for rapid AF detection in pistachios. The system used a xenon chloride (XeCl) excimer laser (308 nm) as the excitation source and a UV–Vis spectrometer for emission capture, targeting the known fluorescence bands of AFs: 425–480 nm (AFB_1_, AFB_2_) and 480–560 nm (AFG_1_, AFG_2_). Pistachio nuts naturally contaminated with *A. flavus* and *A. parasiticus* were tested at different incubation times to attain different AF levels. The fluorescence spectra of pistachio shells showed clear correspondence with contamination levels, and spectral peaks aligned with the characteristic emission of AFs. The approach allowed direct measurement on intact pistachios without peeling, grinding, or complex sample preparation, revealing potential for real-time, non-destructive, industrial-scale screening [116].

**Figure 7:**
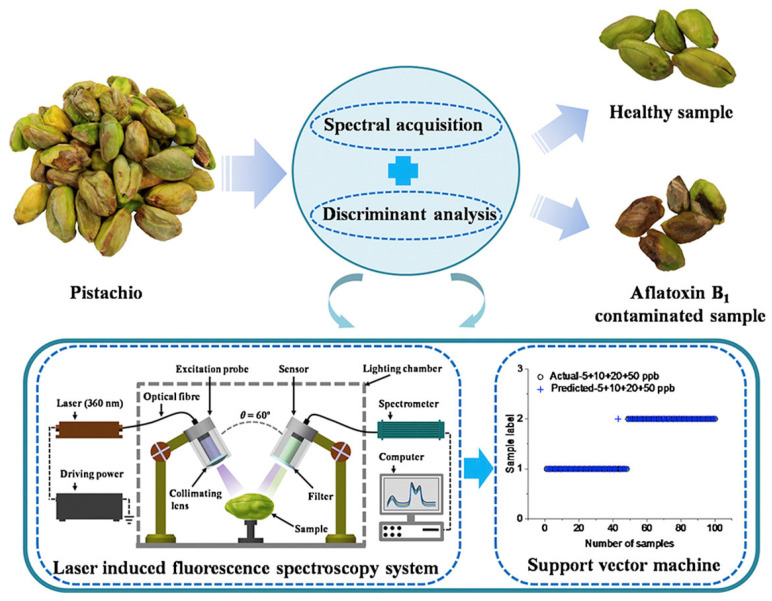
Schematic representation of (LIF) spectroscopy applied to pistachio kernels for AFB_1_ detection. Spectral acquisition combined with discriminant analysis enabled classification of healthy and contaminated samples, with SVM models achieving high accuracy even at low contamination levels (5 ppb). Reprinted with permission from Wu et al. [117]. Copyright© (2019) Elsevier).

Wu et al. [117] investigated the application of LIF spectroscopy for detecting AFB_1_ in pistachio kernels. Using a 360 nm UV laser, emission spectra were recorded from 174 to 1100 nm in two pistachio varieties (“Wanlong” and “Hengkang”) spiked with 5, 10, 20, and 50 ppb AFB_1_ levels. Spectral analysis revealed that contaminated kernels displayed lower fluorescence intensity (mainly in the 410–600 nm region) compared to uncontaminated controls (Figure 7). These spectral differences suggested that LIFS can sensitively capture contamination signatures at levels close to international regulatory thresholds [117].

**Figure 8:**
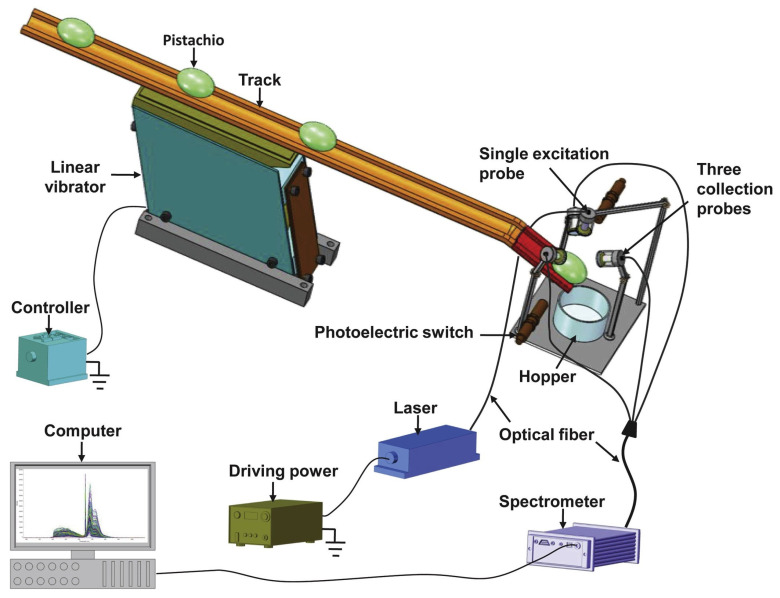
Diagram of the online LIF spectroscopy setup incorporating three probes for monitoring AF contamination in pistachio nuts. Reprinted with permission from Wu and Xu [8]. Copyright© (2020) Elsevier.

Wu and Xu designed and tested an LIF spectroscopy system for online detection of AFB_1_ contamination in pistachio kernels. The setup used a 360 nm excitation laser coupled with three collection probes positioned around the kernel to capture fluorescence emissions across 174–1100 nm (Figure 8). Two pistachio varieties (“Yaoshengji” and “Wanlong”) were artificially contaminated at concentrations of 5, 10, 20, 30, and 50 ppb. Spectral analysis showed clear differences in fluorescence intensity between uncontaminated and contaminated samples, particularly in the 374–609 nm band (linked to AFB_1_ and oxidative products) and 650–800 nm (related to chlorophyll and pigments). The system could detect AFB_1_ at a screening rate of 1 kernel/s, indicating its potential for dynamic, high-throughput industrial applications [8]. These researchers earlier developed a multiplexing fiber optic LIF spectroscopy system to evaluate how different numbers of collection probes affect detection of AFB_1_ contamination in pistachios. Using a 360 nm excitation laser, fluorescence emission spectra were recorded across 174–1100 nm for 300 pistachio kernels artificially contaminated at 5, 10, 20, 30, and 50 ppb. Three detection modes were tested: one-probe, two-probe, and three-probe. The results showed that adding multiple probes improved signal collection, as contaminated kernels exhibited lower fluorescence intensity in the 390–660 nm range compared to controls. The three-probe configuration represented the clearest discrimination, confirming that spatially resolved fluorescence improves sensitivity for AF detection [118].

Magnus et al. [119] have recently advanced LIF spectroscopy for multi-defect detection in pistachios, targeting not only AF contamination but also shells and tree debris. The study compared one-photon induced fluorescence (OPIF, UV excitation at 385 nm) and two-photon induced fluorescence (TPIF, near-infrared (NIR) excitation at 740–770 nm) using a femtosecond pulsed Ti/sapphire laser system. OPIF spectra showed rich emission features between 400 and 750 nm, capturing both AF-associated signals (400–500 nm) and chlorophyll fluorescence (650–700 nm). In contrast, TPIF provided narrower spectral information (600–700 nm) linked mainly to chlorophyll. Since OPIF produced stronger signals and clearer discrimination, it presented superior suitability for practical screening of pistachio kernels, shells, tree parts, and AF-contaminated material [119].

### 5.4. Imaging Sensors

Imaging sensors have been increasingly investigated as non-destructive tools for detecting AF contamination in pistachios. By capturing fluorescence, thermal, or spectral signatures, these methods facilitate rapid screening and classification of contaminated nuts without the need for destructive chemical analysis [26,120]. Lunadei et al. [120] developed an image-based vision system to detect bright greenish yellow fluorescence (BGYF) on pistachios and cashews, a phenomenon strongly associated with AF-producing fungi (*A. flavus* and *A. parasiticus*). The system used a long-wave UV lamp (365 nm) as the excitation source and a charge-coupled device (CCD) camera fitted with optical filters (410–600 nm range) to capture fluorescence images. Image processing algorithms isolated fluorescent stains and classified nuts into two groups: fluorescent stain (FS) and non-fluorescent stain (NFS). Chemical validation using HPLC with immunoaffinity cleanup confirmed that samples classified as FS contained the majority of AF contamination (92% in pistachios and 82% in cashews). Discriminant analysis identified 520 and 480 nm as optimal wavelengths for pistachios, and 440 and 600 nm for cashews, yielding complete separation of FS and NFS groups. Although BGYF is not a definitive indicator of AF, this vision-based method can be integrated into automated sorting systems to provide a fast, non-destructive screening technique for identifying high-risk nuts [120]. Beyond imaging, recent work performed by Rezaee et al., has also explored hybrid sensor systems, such as the integration of machine vision with electronic noses, supporting complementary volatile and visual feature analysis for pistachio AF risk monitoring [24].

Mastrodimos et al. [121] applied thermal and digital red-green-blue (RGB) imaging to monitor *A. flavus* infection in pistachios. Thermal imaging with a Forward-Looking Infrared (FLIR) camera detected surface temperature differences between infected and healthy nuts as early as 6 h post-inoculation, preceding visible mycelium in RGB images (24–48 h). Infected kernels showed localized cooling (+0.5 to +2.0 K), and growth dynamics were modeled with a Weibull distribution, showing distinct rates compared with *A. carbonarius* infection in grapes. While thermal imaging proved useful for early non-destructive detection, RGB imaging was limited by low color contrast between pistachio surfaces and fungal mycelium [121]. Most recently, Williams et al. [26] reported HSI as a non-destructive method to classify pistachios by AF contamination levels. Using a benchtop HSI system (400–1000 nm, 447 spectral channels), a dataset of 300 images was collected across three contamination categories: <8 µg/kg, >160 µg/kg, and >300 µg/kg. Key discriminant wavelengths were identified at 399.98, 584.64, 704.64, 866.21, and 1002.23 nm, indicating potential for replacing costly hyperspectral setups with cheaper multispectral systems. HSI provided clear differentiation of pistachio samples by spectral signatures, indicating its potential for inline food safety monitoring [26].

Biosensing platforms for AF monitoring in pistachios vary widely in terms of detection principle, sensitivity, and applicability. Although electrochemical and optical sensors generally show high sensitivity, differences in analysis time, operational complexity, and cost strongly influence their practical deployment. To contextualize these strengths and limitations, Table 2 presents a comparative overview of representative biosensing platforms, emphasizing their LODs, analysis speed, technical demands, and associated assay or instrumentation costs.

## 6. AI- and ML-Driven Prediction and Control for Aflatoxin Contamination

Advances in chemometrics and ML have allowed powerful new approaches for AF detection in pistachios. By extracting meaningful patterns from spectral and imaging data, these methods can classify contaminated kernels with high accuracy and even quantify toxin levels. Compared with traditional techniques, data-driven models offer greater speed, non-destructive testing, and potential for real-time industrial applications [8,117,122].

To discriminate contaminated from uncontaminated kernels, Wu et al. [117] applied several chemometric and machine learning techniques. Principal component analysis (PCA) provided initial visualization but did not clearly separate groups. Supervised algorithms, including linear discriminant analysis (LDA), logistic regression classification (LRC), and support vector machine (SVM), were examined, combined with spectral pre-treatments such as standard normal variate (SNV) and second derivative filtering. Among these models, the best performance belonged to SVM, with a classification accuracy of 92–100% and false negative errors close to zero, even at contamination as low as 5 ppb. These results showed the value of combining LIF spectroscopy with AI-driven classifiers for reliable pistachio AF screening, although the authors emphasized the need for further validation with naturally contaminated samples [117].

Wu and Xu also applied multiple chemometric and ML approaches to classify and quantify contamination. For discrimination, LDA led to classification accuracies above 91%, with no false negatives at the 5 ppb level as a critical threshold for regulatory compliance. The best performance for quantitative prediction of AFB_1_ content was for partial least squares regression (PLSR), optimized with competitive adaptive reweighted sampling (CARS) for wavelength selection. The CARS-PLS model resulted in a prediction correlation of 0.8903 with a root mean square error of prediction (RMSEP) below 6.20 ppb for pooled samples, confirming its ability to extract key wavelengths (e.g., 428, 520, and 741 nm) associated with contamination. Despite strong laboratory performance, the authors emphasized the need for validation on naturally contaminated kernels and further hardware improvements to handle uneven AF distribution and higher processing speeds [8]. In a distinct study, they re-applied several statistical and ML approaches to classify and predict contamination levels. PCA had partial separation but was insufficient alone. LDA led to an accuracy of >90%, but SVM provided superior performance, with ≥97% accuracy and zero false negatives in the three-probe mode. For quantitative prediction, Stepwise Multiple Linear Regression (SMLR) was superior to PLSR, especially when using the broader 174–1100 nm range. In the three-probe mode, SMLR presented strong calibration/validation correlations (r > 0.95) and low prediction error (RMSEP ≈ 4.4 ppb). Key discriminant wavelengths were identified in the 440–565 nm range, consistent with known AF fluorescence signatures [118]. Magnus et al. [119] also assessed multiple classifiers such as LDA, quadratic discriminant analysis (QDA), and K-nearest neighbors (KNN). Using OPIF spectra and QDA with 10 selected wavelength ratios, the model achieved 99.2% accuracy for healthy kernels, while maintaining a very low false negative rate (0.8%) and a false positive rate of 6.2%. By contrast, TPIF delivered weaker classification performance (≈87% accuracy), reflecting limited spectral information. However, they reported that all AF-contaminated samples were correctly classified across the tested models. The study concluded that OPIF combined with QDA represents a state-of-the-art, non-destructive, high-accuracy approach for industrial pistachio screening [119].

Recent studies show the strong potential of ML in enhancing non-destructive AF detection in pistachios. Williams et al. [26] applied deep learning to HSI data, showing that Residual Networks (ResNet) had the highest performance with 96.7% accuracy at 866 nm, while dimensionality reduction combined with K-Means clustering reached 84.4%. Generative models like variational autoencoders (VAEs) and deep convolutional generative adversarial networks (DCGANs) were also tested. They showed potential for detecting anomalies and creating extra training data, but their performance was limited by the small dataset size [26]. These findings demonstrate that coupling HSI with advanced ML models can substantially improve the accuracy, speed, and scalability of pistachio AF monitoring compared to traditional chemometric approaches.

Apart from spectroscopy and imaging, AI/ML techniques are increasingly applied to predictive modeling of AF risk. Environmental and agronomic variables such as temperature, humidity, rainfall, irrigation practices, and storage conditions are strong determinants of *Aspergillus* growth and AF accumulation. Algorithms including random forests, gradient boosting, artificial neural networks (ANNs), and recurrent neural networks (RNNs) have been used in cereals and peanuts to forecast contamination hotspots with high accuracy [123,124,125]. For pistachios, such models could be integrated into early warning systems that alert producers or exporters to high-risk batches before they reach international markets, reducing costly rejections. Accordingly, the predictive capacity of these models improves when combined with real-time sensor data, making them adaptable for dynamic risk management [124].

AI is emerging as a powerful tool for advancing biosensor platforms. Signal interpretation remains a bottleneck for many electrochemical and optical AF sensors, where matrix interference and noise can obscure binding signals. ML classifiers, including SVMs and ensemble models, can be trained to recognize subtle AF-specific patterns, improving sensitivity and lowering false positive rates. For portable or field-deployable devices, this integration allows more robust and reliable operation in complex pistachio matrices. Furthermore, deep learning–based feature extraction can accelerate calibration, providing semi-automated operation that reduces the need for highly trained technicians [126].

Another emerging application is the use of AI for automated kernel sorting and defect recognition. Convolutional neural networks (CNNs) applied to RGB, multispectral, or hyperspectral images can identify kernels with surface defects, fungal growth, or discoloration associated with AF risk [26]. This approach not only aids in removing contaminated kernels but also increases the overall efficiency of optical sorting lines in pistachio processing facilities. When combined with chemometric models, AI-driven sorting systems offer a strong complement to chemical or sensor-based detection.

Finally, AI/ML can be embedded into decision support systems that create a closed-loop safety framework. By processing biosensor or imaging data in real time, algorithms can assess whether contamination thresholds are exceeded and automatically trigger appropriate nonthermal interventions such as cold plasma or pulsed light. Such adaptive control minimizes processing costs while ensuring regulatory compliance and quality preservation. In the longer term, integration of AF risk modeling, biosensing, and nonthermal decontamination into unified AI-driven platforms could transform PSM from reactive testing to proactive, intelligent control [124].

**Figure 9:**
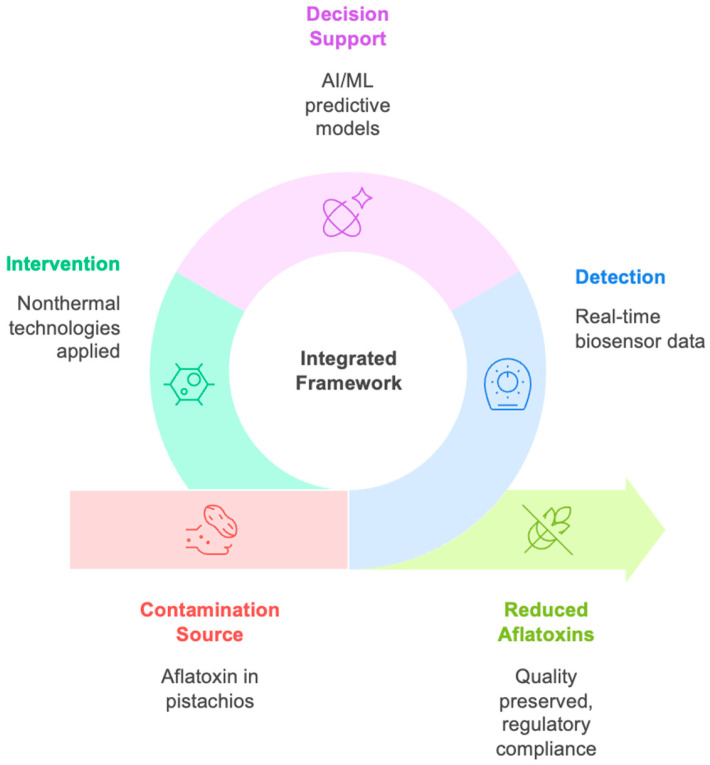
Conceptual framework illustrating the integration of real-time biosensing, AI/ML-driven decision support, and nonthermal decontamination strategies for AF control in pistachios.

Nonthermal decontamination, biosensing platforms, and AI/ML models, although reviewed separately, achieve their greatest value when integrated into a unified framework. Real-time AF data generated by biosensors can be processed by AI/ML-driven decision support systems to predict contamination risks and assess when intervention thresholds are exceeded. These outputs can then guide or optimize nonthermal technologies such as cold plasma or PL, enabling efficient AF reduction while preserving product quality. Such a closed-loop approach demonstrates the potential of combining biosensing, AI/ML, and nonthermal interventions to deliver intelligent and sustainable PSM (Figure 9).

## 7. Conclusions and Future Directions

This review presented the critical challenge of AF contamination in pistachios and summarized the advances in innovative strategies to mitigate and monitor this risk. Conventional decontamination methods such as roasting, irradiation, and acid/alkaline treatments have demonstrated partial detoxification but often compromise nutritional and sensory quality. Emerging nonthermal technologies, including PL, cold plasma, and nanomaterial-based adsorption or packaging, show considerable performances by effectively reducing *A. flavus* growth and AF levels with maintaining pistachio quality parameters. Moreover, biosensing platforms, including electrochemical immunosensors, aptasensors, and fluorescence/imaging-based devices provided rapid, portable, and selective detection of AFs in pistachios, meeting strict regulatory limits. Furthermore, AI and ML approaches have advanced predictive modeling of fungal growth and AF biosynthesis, presenting scalable solutions for real-time monitoring and early risk detection throughout supply chains. This progress emphasizes the synergistic potential of combining nonthermal reduction strategies, advanced biosensors, and AI-driven analytics into integrated systems for ensuring pistachio safety and global trade compliance. Based on current evidence and technology readiness, CAP and PL are the two nonthermal technologies closest to broad commercial adoption in pistachio processing, due to short treatment times, conveyor compatibility (PL), residue-free operation (CAP), and growing pilot-scale demonstrations.

Future research should focus on optimizing nonthermal decontamination processes such as cold plasma, PL, and irradiation to obtain higher detoxification efficiencies with minimal impact on nutritional and sensory attributes. For cold plasma, studies should systematically define optimal gas composition (air/oxygen/argon and humidity), discharge power density, electrode–product gap, and exposure time to maximize AF degradation while minimizing lipid oxidation, color loss, and protein solubility changes; standardized dose/fluence metrics and in-kernel dose mapping should be reported. For PL/UV, critical parameters to optimize include pulse energy, emission spectrum, lamp–kernel geometry, conveyor speed, and fluence uniformity on irregular or in-shell kernels to reduce shadowing and ensure consistent treatment. For irradiation (γ/e-beam/X-ray), optimization should balance dose and dose-rate with sensory endpoints, while characterizing degradation product profiles and toxicology to support regulatory acceptance. Integrating these methods with natural bioactive compounds, microbial biocontrol agents, and nanomaterial-based coatings or packaging can provide multi-barrier protection against *A. flavus* infection and AF accumulation. Moreover, scaling laboratory findings into pilot- and industrial-scale systems remains a priority, particularly by assessing energy efficiency, cost-effectiveness, and compliance with food safety standards. Next-generation biosensors for pistachio monitoring should emphasize miniaturization, portability, and integration into automated sorting and processing lines. Research should expand on hybrid devices combining electrochemical, optical, and aptamer-based mechanisms, reinforced with nanomaterials such as MXenes, MOFs, or conductive polymers, to push LODs below current regulatory thresholds. Long-term stability, multiplexing capacity for multiple mycotoxins, and smartphone or Internet of things (IoT)-enabled interfaces will be critical for real-world adoption, particularly in resource-limited contexts. Priority biosensor metrics should include matrix-matched LODs below legal limits, < 5% signal drift over 8 h runs, anti-fouling surface chemistries, calibration transfer across instruments, and ruggedization for dust/oil exposure; inline links to sorting ejectors and CAP/PL units should enable automatic hold-and-release decisions. AI and ML should be scrutinized to link multi-sensor datasets, including HSI, fluorescence, Raman, and electronic nose outputs, with predictive models of fungal growth and AF biosynthesis. Larger datasets from naturally contaminated pistachios are essential to strengthen model robustness and reduce false negatives at critical contamination thresholds. Models should be trained with class-imbalance handling and externally validated across orchards and seasons; success criteria should emphasize very low false-negative rates at regulatory thresholds and interpretable feature importance to support process control. Future developments should also focus on lightweight algorithms for real-time decision-making, cloud-based monitoring platforms, and digital twins for pistachio processing lines, driving predictive and adaptive control of contamination risks across the supply chain. Lightweight, on-edge architectures (e.g., quantized CNNs or gradient-boosting ensembles) with late-fusion of HSI/fluorescence/Raman/e-nose signals are recommended for embedded deployment. Overall, an AI-supervised, sensor-driven CAP/PL intervention loop represents a feasible near-term blueprint for proactive pistachio safety management and reduced export rejections.

## Figures and Tables

**Table 1:** Comparison of nonthermal methods for AF reduction in pistachios.

Method	Efficacy (AF Reduction, %)	Impact on Product Quality	Scalability/TRL *	Cost/Practicality	Ref.
Pulsed light (PL)	Up to 93–98% in solutions; 22–58% on pistachios depending on dose/time	Minimal chemical changes; Shadowing decreases effectiveness on irregular kernels	Medium TRL (=6–8); Suitable for conveyor integration	Moderate; Requires line-of-sight engineering	[15]
Ozone	AFB_1_/AFG_1_ are more sensitive than AFB_2_/AFG_2_; Effective across nuts	Risk of lipid oxidation and sensory changes at high doses	High TRL (=9); Already used in food industry	Relatively low-cost; Process control required	[15,36]
Cold atmospheric plasma (CAP)	Up to ~99% AFB_1_/AFG_1_; ~4–5 log *Aspergillus* reduction	Minimal heating; Residue-free; Matrix effects may reduce efficacy	Medium to high TRL (=5–7); Requires standardization and scale-up validation	Moderate; Equipment cost but efficient treatment	[103]
Nanomaterial Adsorption/Packaging	Selective binding of AFs (e.g., polydopamine sorbents, silver nanoparticles-loaded films)	Non-destructive; Potential migration/regulatory issues	Low TRL (=2–4); Emerging, Needs industrial validation	Unclear cost; Regeneration/migration challenges	[104]

* Technology Readiness Level (TRL): TRL 1–3 → Early-stage research, proof-of-concept, TRL 4–6 → Lab validation and pilot-scale testing, TRL 7–8 → Demonstration and pre-commercial scale, and TRL 9 → Fully implemented in industry/commercial deployment. This classification was originally developed by the National Aeronautics and Space Administration (NASA).

**Table 2:** Comparison of recent biosensing platforms for AF detection in pistachios.

Platform (Target) *	Matrix	LOD	Analysis Time **	Complexity **	Relative Cost	Ref.
Electrochemical immunosensor (AFB_1_)	Buffer; Spiked pistachio	0.5 ng/mL (buffer); 1.0 ng/mL (pistachio)	n.r.	Moderate (SPE + EIS)	Moderate	[20]
Electrochemical immunosensor (MOF/MXene–EIS, AFB_1_)	Spiked pistachio	0.008 ng/mL	n.r.	Moderate-High	Moderate	[22]
Competitive electrochemical immunosensor (SPCE + HRP, Total AFs)	Buffer; pistachio (with IAC)	0.017 µg/L (buffer); 0.066 µg/kg (pistachio)	n.r. (includes IAC step)	High	Moderate-High	[7]
Aptasensor (GO/AuNW–DPV, AFB_1_)	Spiked & naturally contaminated pistachio	1.4 pM	n.r.	Moderate	Low-Moderate	[114]
Aptasensor (PAni–EIS, AFB_1_)	Real foods incl. pistachio	10 pM	~30 min	Low-Moderate	Low	[115]
THz metamaterial sensor (AFB_2_)	Solution	7.28 × 10^−11^ mg/mL; 4.19 × 10^−9^ mg/mL; 1.22 × 10^−7^ mg/mL (low/medium/high concentration models)	n.r.	High	High	[19]

* MOF/MXene–EIS: Metal–organic framework/MXene-based electrochemical impedance spectroscopy; SPCE + HRP: Screen-printed carbon electrode with horseradish peroxidase; GO/AuNW–DPV: Graphene oxide/gold nanowire-based differential pulse voltammetry; PAni–EIS: Polyaniline-based electrochemical impedance spectroscopy; THz-TDS: Terahertz time-domain spectroscopy. ** n.r.: not reported, IAC: immunoaffinity column, SPE: gold screen-printed electrode, EIS: electrochemical impedance spectroscopy.

## Data Availability

Data is contained within the article.

## Abbreviations

The following abbreviations are used in this manuscript:

AFB_1_	Aflatoxin B_1_
AI	Artificial Intelligence
ANNs	Artificial Neural Networks
AuNW	Gold Nanowire
BGYF	Bright Greenish Yellow Fluorescence
BSA	Bovine Serum Albumin
CAP	Cold Atmospheric Plasma
CARS	Competitive Adaptive Reweighted Sampling
CCD	Charge-Coupled Device
CMD	Carbo-Methyldextran
CNNs	Convolutional Neural Networks
DBD	Dielectric Barrier Discharge
DCGANs	Deep Convolutional Generative Adversarial Networks
dpi	Days Post Inoculation
DPV	Differential Pulse Voltammetry
EBI	Electron Beam Irradiation
EIS	Electrochemical Impedance Spectroscopy
ELISA	Enzyme-Linked Immunosorbent Assay
EW	Electrolyzed Water
FDA	Food and Drug Administration
FLD	Fluorescence Detection
FLIR	Forward-Looking Infrared
GO	Graphene Oxide
GRAS	Generally Recognized As Safe
HPLC	High Performance Liquid Chromatography
HRP	Horseradish Peroxidase
HSI	Hyperspectral Imaging
IAC	Immunoaffinity Column
IARC	International Agency for Research on Cancer
IoT	Internet of Things
LDA	Linear Discriminant Analysis
KNN	K-Nearest Neighbors
LIF	Laser-Induced Fluorescence
LRC	Logistic Regression Classification
ML	Machine Learning
MCNPX	Monte Carlo N-Particle eXtended
MS	Mass Spectrometry
NASA	National Aeronautics and Space Administration
(N)FS	Non-Fluorescent Stain
NIR	Near-Infrared
OPIF	One-Photon Induced Fluorescence
PAni	Polyaniline
PCA	Principal Component Analysis
PGCC	Persian Gulf Cooperation Council
PL	Pulsed Light
PLSR	Partial Least Squares Regression
PSM	Pistachio Safety Management
QDA	Quadratic Discriminant Analysis
RASFF	Rapid Alert System for Food and Feed
ResNet	Residual Networks
RGB	Red-Green-Blue
RMSEP	Root Mean Square Error of Prediction
RNNs	Recurrent Neural Networks
RSD	Relative Standard Deviation
SERS	Surface-Enhanced Raman Scattering
SMLR	Stepwise Multiple Linear Regression
SNV	Standard Normal Variate
SP(C)Es	Screen-Printed (Carbon) Electrodes
SVM	Support Vector Machine
THz-TDS	Terahertz-Time-Domain Spectroscopy
TLC	Thin Layer Chromatography
TPIF	Two-Photon Induced Fluorescence
TRL	Technology Readiness Level
VAEs	Variational Autoencoders
